# What are the outcomes of core decompression without augmentation in patients with nontraumatic osteonecrosis of the femoral head?

**DOI:** 10.1007/s00264-020-04790-9

**Published:** 2020-09-04

**Authors:** Octavian Andronic, Ori Weiss, Haitham Shoman, Philipp Kriechling, Vikas Khanduja

**Affiliations:** 1 Department of Orthopaedics, Balgrist University Hospital, Forchstrasse 340, 8008 Zürich, Switzerland; 2 Young Adult Hip Service, Department of Trauma and Orthopaedics, Addenbrooke’s - Cambridge University Hospitals NHS Foundation Trust, Hills Road, Cambridge, CB2 0QQ UK; 3 Department of Orthopaedic Surgery, Meir Medical Center, Kfar-Saba, Israel; 4 Department of Global Health and Social Medicine, Harvard Medical School, Boston, MA USA

**Keywords:** Core decompression, Hip preservation, Hip arthroscopy, Avascular osteonecrosis of the femoral head, AVN, Osteonecrosis

## Abstract

**Purpose:**

Core decompression (CD) of the femoral head is performed to preserve the hip in avascular necrosis (AVN). The outcome following this procedure differs based on the medical centre and the technique. Also, the time to total hip replacement (THR) and the percentage of patients subsequently undergoing a THR are controversial.

**Methods:**

A systematic review was performed following PRISMA guidelines. The search included CENTRAL, MEDLINE, EMBASE, Scopus, AMED and Web of Science Core Collection databases. Studies reporting the outcome of CD for AVN were assessed. Studies using additional implants, vascularized grafts or any type of augmentation were excluded. Quality assessment was performed using the Joanna Briggs Institute Critical Appraisal Checklist (JBI CAC) tool.

**Trial registration:**

International prospective register of systematic reviews (PROSPERO) - CRD42018100596.

**Results:**

A total of 49 studies describing 2540 hips were included. The mean weighted follow-up time was 75.1 months and the mean age at surgery was 39 years. Twenty-four of 37 studies reported improvement in all outcome scores, whilst 9/37 studies report only partial improvement post-operatively. Four studies (4/37) described poor clinical outcomes following intervention. Data was pooled from 20 studies, including 1134 hips with a weighted mean follow-up of 56 months. The percentage of hips undergoing THR averaged 38%. The time to THR had a weighted mean of 26 months after CD.

**Conclusion:**

Pooled results from 1134 hips and of these nearly 80% with early stage of osteonecrosis, showed that approximately 38% of patients underwent a total hip replacement at an average of 26 months following core decompression without augmentation.

**Electronic supplementary material:**

The online version of this article (10.1007/s00264-020-04790-9) contains supplementary material, which is available to authorized users.

## Introduction

Osteonecrosis or avascular necrosis (AVN) of the femoral head is a challenging condition that ultimately leads to patients undergoing a total hip replacement (THR) [1]. These patients are young and therefore usually require further revision hip replacements and multiple surgical procedures [2]. The aetiology for AVN is varied and includes a variety of conditions that lead to a compromised blood supply of the femoral head. These include oral corticosteroids, excessive alcohol consumption, Gaucher disease, sickle cell anemia, trauma, thrombosis and systemic lupus erythematosus and in a large proportion of patients, a cause cannot be established and is therefore termed idiopathic [3]. Furthermore, the staging systems for progression of disease are different across the literature and pose a significant challenge in stratifying disease, defining surgical indications and establishing outcomes [4]. The most common classification systems in use are Ficat [5] /Modified Ficat [6], University of Pennsylvania/Steinberg [4] and ARCO (Association Research Circulation Osseous) [7–9].

Core decompression is a surgical intervention that is used early in the disease process. The procedure potentially decreases the intraosseous pressure in the femoral head, relieves pain and reestablishes blood flow helping healing of the necrotic fragment. Multiple augmentation techniques with core decompression have also been described and seem to further improve outcomes [10, 11].

However, the eventual outcome and time to THR remains controversial [12–14]. It is also not clear whether a mechanical decompression in the form of core decompression alone is sufficient and efficient enough in all stages of AVN to prevent progression and delay the need for a THR.

The purpose of this study, therefore, was to assess the outcomes and time to THR following core decompression of the femoral head without any augmentation for non-traumatic AVN.

## Materials and methods

### Search strategy and criteria

Two reviewers (OA and OW) searched the online databases (CENTRAL (Cochrane Central Register of Controlled Trials), MEDLINE, EMBASE, Scopus, AMED and Web of Science Core Collection) for literature describing the outcomes of core decompression without augmentation for non-traumatic AVN of the femoral head. A total of eight combinations of the following keywords were used: “femoral head” with “osteonecrosis”, “avascular necrosis”, “aseptic necrosis”, “avn” with the terms - “core decompression” or “surgery”. The Preferred Reporting Items for Systematic Reviews and Meta-Analyses (PRISMA) guidelines were used for designing this study. All published studies from inception until January 1, 2020, were included in the systematic search. The protocol of this systematic review has been registered in the international prospective register of systematic reviews (PROSPERO) under the registration number CRD42018100596 and been published recently [15].

### Study screening/data abstraction

A detailed search strategy and the inclusion and exclusion criteria are shown in Table 1. Both the reviewers independently abstracted the relevant study data from the final pool of included articles and recorded this data on a spreadsheet designed *a priori*. Participant-specific demographics extracted from each study included number of hips, age, gender, body mass index (BMI), presumed primary aetiology, stage of disease, surgical technique, clinical outcome (with preoperative and postoperative results where applicable), radiological outcome, time to joint replacement (THR), average follow-up and specific comments (if any).

**Table 1:** Study selection criteria

Inclusion criteria	Exclusion criteria
•Human studies in English language from inception until January 1, 2020	•Non-English articles
•Minimum level IV case series studies using Oxford Centre for Evidence-Based Medicine 2011 Levels of Evidence	•Review/hypothesis/technique articles/oral presentations or cadaveric/animal studies
•Established diagnosis of avascular necrosis of the femoral head, outcomes together with decompression technique were reported	•Studies including patients who underwent previous surgery
•At least 10 hips were evaluated	•Patient population with sickle cell disease
•Patients were classified either based on aetiology or on the stage of the disease: Ficat/Modified Ficat or University of Pennsylvania/Steinberg or ARCO	•Any type of augmentation was used (e.g. vascularized bone grafts or bone marrow stem cells) •Studies including patients with associated trauma or labral tears

*ARCO* Association Research Circulation Osseous

### Data extraction and quality assessment

The quality of the RCTs was evaluated as per the guidance of the Cochrane Risk of Bias assessment tool. The quality of all the studies was then assessed using the Joanna Briggs Institute Critical Appraisal Checklist (JBI CAC) [16]. A scoring system was then used per study such as studies that answered yes to a question from the checklist scored 2, not clear scored 1 and no scored 0. Each score was then converted into a percentage to harmonize the scoring system.

### Data analysis and synthesis

Statistical analyses were performed using SPSS (IBM SPSS Statistics, Version 24.0; Chicago, Illinois) and Graphpad Prism (Graphpad Software, Version 8; San Diego, California).

In order to explore heterogeneity and evaluate studies based on possible confounders, forest plots were developed for calculation of effect size and confidence intervals (95%). For proportions of hips undergoing hip replacement, the datasets were developed from calculated individual proportions of studies and their confidence intervals. A random effect model was used. Heterogeneity was calculated using Comprehensive Meta-Analysis v2 (CMA), NJ, 07631, USA. According to all of included studies, the *α* level was set at 0.05, and all *p* values were two-tailed.

### Interpretation of the forest plots

Forest plots were presented to summarize the data (Fig. 2). Each horizontal line on a forest plot represents a case series included in the analysis. The length of the line corresponds to a 95% CI of the corresponding case series’ effect estimate. The effect estimate is marked with a solid square. The size of the square represents the weight that the corresponding study exerts in the analysis. The *I*^2^ value represents the calculated heterogeneity. Values less than 50% represent mild to moderate heterogeneity, whereas values greater than 50% represent substantial to considerable heterogeneity.

## Results

### Search results and demographics

**Fig. 1:**
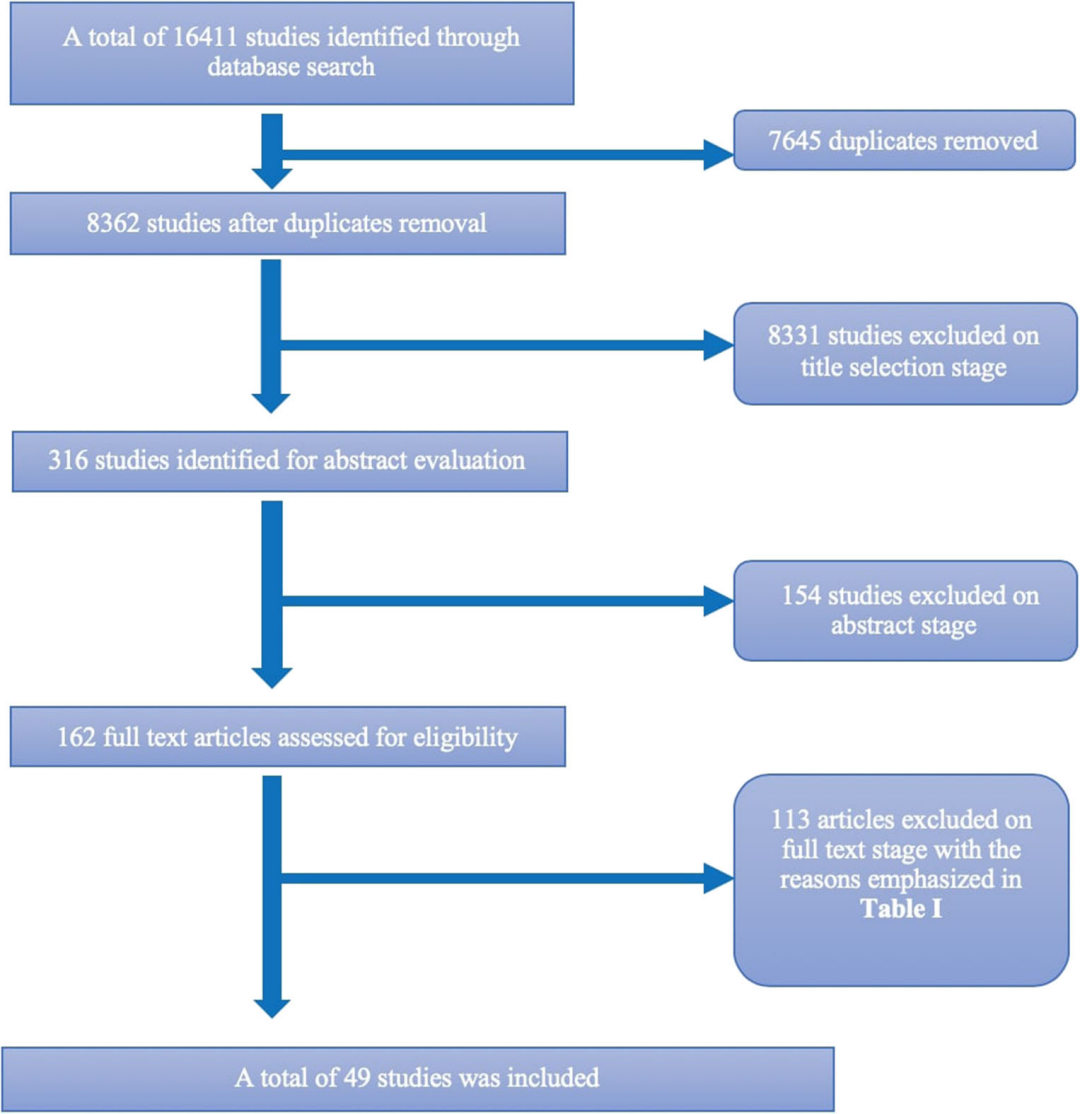
Flowchart of the systematic search

The initial search yielded a total of 16411 studies. After removing duplicates, there were 8362 articles. These were then screened for eligibility against the inclusion and exclusion criteria (Table 1) and finally, 49 articles [24–70] were included for the full-text review and definitive analysis (Fig. 1). The reasons for exclusion were noted and are described separately in Suppl. Table 1.

A total of 2540 hips were included in the study. There were 1122 males (61.5%) and 702 females (38.5%). The mean weighted follow-up time was 75.1 months and the mean age at surgery of patients was 39 years. The main aetiologies of AVN included the following: usage of corticosteroids (53.5% of patients), idiopathic (23.1% of patients) and alcohol abuse (22.5% of patients) (Suppl. Table 2). The techniques used for drilling during core decompression were varied. These included simple or multiple drilling using different instruments of different diameters (trephine, cannulated drills, K-wires and Steinman pins with diverse diameters).

### Classification systems

An accentuated heterogeneity was found among the classification systems for staging of the disease. Majority of the studies, 20 (40%), used the original “Ficat” classification [24, 27, 29, 32, 34, 36, 41, 46, 48–52, 56–59, 61, 62, 67]. Ten studies (20%) followed the “Modified Ficat” classification [12, 25, 31, 44, 47, 54, 55, 63, 65, 70] and other eleven (22%) [26, 28, 30, 37–39, 45, 60, 66, 68, 69] used the ARCO system. Finally, nine studies (18%) [12, 13, 33, 35, 40, 42, 43, 53, 64] applied the “Steinberg/University of Pennsylvania” classification. There was a single study that reported the hips separately using two classification systems (Modified Ficat/Steinberg) [12].

### Quality assessment

Quality assessment of the 49 studies revealed that there were 27 level IV studies (case series), 12 level III studies, seven level II studies and three level I studies (RCTs—randomized controlled trials). The Joanna Briggs Institute Critical Appraisal Toolkit (JBI-CAT) included the assessment of methodology and of the reported risk of bias (Suppl. Table 2). The studies averaged a score of 82%, which is an indicator of good quality with the majority of studies scoring 75% or more (37/49).

### Clinical and radiological outcome

Thirty-seven out of 49 studies (76%) reported data on clinical outcome. Various tools were used for assessment of outcomes in these studies: Merle d’Aubigné-Postel, VAS (visual analogue score), Harris hip score (HHS), WOMAC, SF36-Physical, SF36-Mental, Lequesne Index and pain rating index (PRI). There was an obvious lack of a unified reporting tools which required further simplification to interpret the results. As such, the outcomes were simplified down to a binary level: clinical improvement yes or no and radiological progression: yes or no. Post-operative clinical improvement was considered when there was any post-operative improvement reported in the outcome scores. From these, 24/37 studies reported improvement in all outcome scores, whilst 9/37 had only partially achieved better scores post-operatively. Four studies (4/37) described poor outcomes post-operatively. Time to clinical deterioration was reported in 51% (25/49) of the studies, usually corresponding to the time to a THR.

Due to the lack of separate stratification of pre-operative and post-operative radiological stages in the selected studies, a meaningful statistical summary could not be outlined. Therefore, only a descriptive analysis of the individual studies was performed (Suppl. Table 3–6). The post-operative staging usually mixed the entire cohort of patients, making it impossible to determine which hips did not progress to a THR and which did. However, there were some studies that reported the amount or percentage of hips that did not deteriorate radiologically and these have been recorded in Suppl. Table (3–6).

### Total hip replacement

Both percentage and time to THR following core decompression were documented in 20/49 studies and included a total of 1134 hips (Suppl. Table 7). The pre-operative staging included 890/1134 (78.5%) of hips with early stages of avascular necrosis and no signs of collapse: Ficat classification (6 studies): stage I and II—180/196, stage III and IV—16/196; modified Ficat classification (4 studies): stage I and II—217/300, stage III and IV—83/300; Steinberg classification (3 studies): stage I + II + III—299/402, stage IV + V + VI—103/402; ARCO classification (7 studies): stage I and II—194/236, stage III and IV—42/236.

At the final follow-up with a weighted average of 56 months, 431/1134 (38%) hips were converted to a THR at a calculated weighted average time of 26.3 months (Suppl. Table 7).

**Fig. 2:**
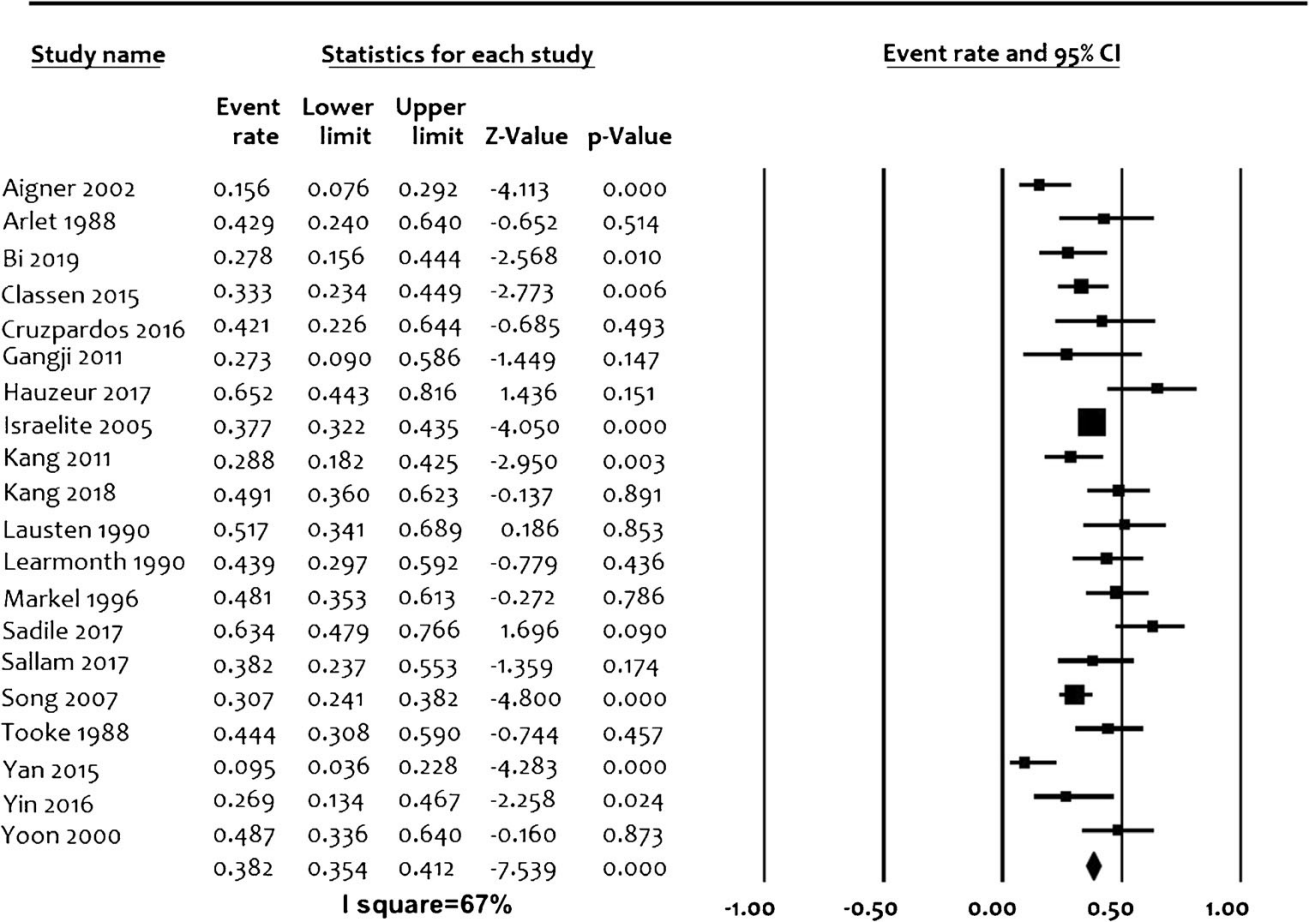
Proportion forest plot of studies reporting percentage of hips undergoing total hip replacement after core decompression. Event—conversion to THA (total hip arthroplasty). The size of the square represents the weight that the corresponding study exerts. *I*^2^—value of calculated heterogeneity

**Fig. 3:**
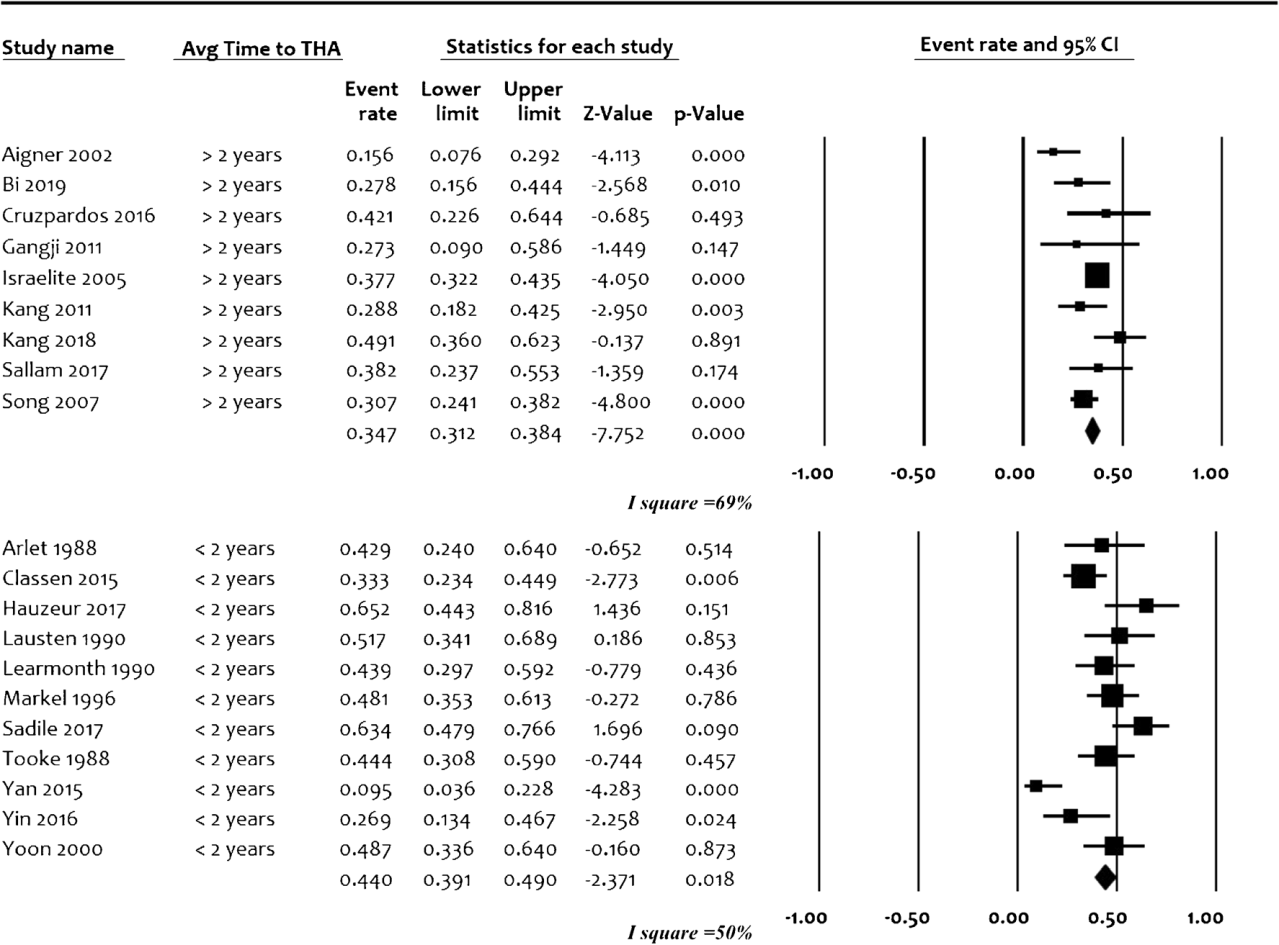
Forest plot differentiating studies with early conversion to total hip arthroplasty (average time to THA < 24 months) versus late conversions (average time to THA > 24 months). Event—conversion to THA (total hip arthroplasty). The size of the square represents the weight that the corresponding study exerts. *I*^2^–value of calculated heterogeneity

The pooled proportion of hips undergoing total hip replacement was 38% (95% confidence interval with lower limit of 35.3% to upper limit of 41.1%) from 20 studies with a total of 1134 cases. There was statistical significance regarding heterogeneity (*I*^2^ value > 80%, *p* < 0.0001) showing the inconsistency of methodological aspects between the studies included in the analysis, which did not allow a detailed meta-analysis (Fig. 2). For further stratification, studies were separately evaluated based on the most probable confounders: inclusion of post-collapse stages in the study population (Suppl. Fig. 1), design (prospective/retrospective) (Suppl. Fig. 2) and average time to total hip replacement (early < 24 months and late > 24 months) (Fig. 3). This could not, however, significantly reduce the heterogeneity, as all *I*^2^ values were above 60%, which may represent substantial heterogeneity regardless of the abovementioned stratification efforts.

## Discussion

Osteonecrosis of the femoral head is a devastating condition for the young adult. Depending on severity of the disease, these patients may require a THR at a particularly young age and are, therefore, likely to require a revision and perhaps a re-revision of their prosthesis at some point in their lives [17]. Early diagnosis, prompt intervention and refining of joint-preserving procedures are therefore especially important in order to avoid or at least delay the need for joint replacement in this unique cohort. Deciding the best treatment algorithm for a young and active patient presenting with avascular necrosis can be challenging. It should commence with selecting a good classification system from the many available that would offer accuracy in evaluating disease progression and stratifying the disease. The optimal treatment of the pre-collapse stage of (ARCO stage 1 and 2) avascular necrosis is controversial. Core decompression is the most commonly performed procedure for treating pre-collapse osteonecrosis, as it has also been shown to be the only cost-effective technique [18, 19]. Increased intramedullary pressure is considered to be at the root cause of the pathophysiology, as it is thought to potentially block off the perfusion to the head of the femur. CD works by drilling one or multiple tunnels from the greater trochanter, through the femoral neck, and into the subchondral bone of the femoral head thereby reducing the intramedullary pressure, promoting blood supply and allowing the necrotic segment to heal [20].

Our study shows that core decompression provides only short-term clinical improvement and partial or complete pain relief in most of the cases (33 out of 37 studies reported post-operative clinical improvement). It should however be noted that reduction of pain may be due to temporary reduction of weight-bearing during the rehabilitation phase and further trials evaluating this aspect are necessary. Our results also demonstrates that approximately 38% of patients underwent a total hip replacement at an average of 26 months following core decompression without augmentation in a large and diverse population with AVN of the femoral head of varied aetiology. This review, however, could not determine whether core decompression alone can arrest disease progression due to lack of stratification and heterogeneity of data.

Our study reveals that the risk of conversion to a THR is fairly high in the shorter term with core decompression alone. It remains to be seen whether augmentation procedures can better these results and obviate the need for a THR in this cohort of patients.

Previous studies looking at the outcomes following core decompression either have gaps in the inclusion and exclusion criteria, are limited by the number of cases included or have mixed all augmentation techniques with core decompression which makes it difficult to interpret the outcomes of core decompression alone as a surgical intervention [21–23]. Our study represents an effort to summarize all the available evidence, which describes core decompression of the femoral head as a sole procedure without additional augmentation, e.g. bone marrow grafting.

There are limitations of our study and most of them are directly linked to the individual limitations of the included studies and heterogeneity of data. The reporting systems were highly variable, from different clinical scores HHS/D’Aubigne/VAS to differing classifications used for staging disease (Ficat or its modification, Steinberg, ARCO). Furthermore, the concept of “procedural success” was not absolute. Whilst most studies considered the absence of radiological progression to be the main finding that suggested success, other authors interpreted clinical improvement as being a success, even in the presence of radiographic deterioration. Not reporting the outcome separately for every single stage subgroup was the biggest challenge in assessment of radiological outcome. Most studies reported the distribution of pre-operative and post-operative radiological stages, without specifically describing which hips actually deteriorated, making it tedious to track longitudinal change for each hip. As such, no conclusions could be made regarding the implications of the preoperative area of osteonecrosis. Also, the lack of granularity and the presence of significant heterogeneity in the data analysed did not allow stratification of outcome based on each specific aetiology (idiopathic/corticosteroids/alcohol abuse or other), as the post-operative outcome was reported cumulative for all patients. This is applied not only to clinical data or radiographic staging but also to conversion rates to THA, which could not be extracted for each aetiology separately.

However, despite these limitations, the strengths of our study are represented by the large patient pool and by the rigorous exclusion criteria that was used. The collateral influence of aetiology (traumatic), systemic disease (sickle cell crisis) or technique heterogeneity (presence of augmentation or bone grafts) was excluded. Also, there was a tenacious stratification based on stage of the disease even in the presence of a variety of classification systems which makes this study unique.

A significant amount of work has been done recently by the ARCO group [7–9] to define the aetiology and arrive at a consensus statement to revise the ARCO classification. Going forward, this classification should be used universally, along with a specific criterion for defining “procedural success” to allow future studies to compare results and avoid heterogeneity in data.

Despite a high degree of heterogeneity amongst studies, core decompression alone achieved short-term clinical improvement in majority of the cases. Pooled results from 1134 hips and of these nearly 80% with early stage of osteonecrosis, showed that approximately 38% of patients underwent a total hip replacement at an average of 26 months following core decompression without augmentation. Future studies should report outcome by stratifying it based on pre-operative stages as proposed by the ARCO group and post-collapse stages of osteonecrosis should be excluded.

## Electronic supplementary material

Suppl. Table 1 (DOCX 16 kb)

Suppl. Table 2 (DOCX 72 kb)

Suppl. Table 3 (DOCX 31 kb)

Suppl. Table 4 (DOCX 26 kb)

Suppl. Table 5 (DOCX 39 kb)

Suppl. Table 6 (DOCX 29 kb)

Suppl. Table 7 (DOCX 41 kb)

Supplementary Figure 1 Forest plot differentiating studies that included patients with preoperative post-collapse stages of osteonecrosis from those that did not. Event – conversion to THA (total hip arthroplasty). The size of the square represents the weight that the corresponding study exerts. I^2^ – value of calculated heterogeneity (JPG 727 kb)

Supplementary Figure 2 Forest plot differentiating results from retrospective versus prospective studies. Event – conversion to THA (total hip arthroplasty). The size of the square represents the weight that the corresponding study exerts. I^2^ – value of calculated heterogeneity (JPG 841 kb)
