# Supplementary material for: What are the outcomes of core decompression without augmentation in patients with nontraumatic osteonecrosis of the femoral head?
Source: Int Orthop. 2020 Sep 4;45(3):605–13. doi: 10.1007/s00264-020-04790-9 (PMC7892522; doi:10.1007/s00264-020-04790-9)
Supplement: Supplementary file 1 — (DOCX 16 kb) [file 264_2020_4790_MOESM1_ESM.docx]

| **Supplementary Table 1.** Reasons for exclusion on the full text assessment stage |
| --- |
| - 26 studies – additional augmentation was used |
| - 20 studies – not in English |
| - 17 studies – full text could not be found or accessed |
| - 15 studies – included traumatic causes of AVN |
| - 11 studies – included subjects with sickle cell disease |
| - 10 studies – poster/oral presentation/review/letter/surgical technique |
| - 6 studies – no CD, only hormonal therapy |
| - 3 studies – no outcome or less than 10 hips were reported |
| - 3 studies – classification system not within inclusion criteria |
| - 2 studies – porous tantalum or other implants were used |
| Total: 113 studies excluded |
