# Supplementary material for: What are the outcomes of core decompression without augmentation in patients with nontraumatic osteonecrosis of the femoral head?
Source: Int Orthop. 2020 Sep 4;45(3):605–13. doi: 10.1007/s00264-020-04790-9 (PMC7892522; doi:10.1007/s00264-020-04790-9)
Supplement: Supplementary file 2 — (DOCX 72 kb) [file 264_2020_4790_MOESM2_ESM.docx]

**Supplementary Table 2.** Demographics of Included Studies

| **Author (Year, Country)** | **Nr.** | **Avg Follow-up (months)** | **Level of Evidence** | **Joanna Briggs Score** | **Surgical Technique** | **Avg Age (years)** | **Sex**  **(No; %)** | **Etiologies**  **(%)** |
| --- | --- | --- | --- | --- | --- | --- | --- | --- |
| Aaron 1989[24] | 50 | 38 | Level III | 95% | Core decompression (Biopsy Trephine) | 43 | 16 (42%) M  22 (58%) F | 71% Corticosteroids, 29% idiopathic |
| Abrisham 2013[25] | 37 | 24 | Level IV | 95% | Core Decompression (needle) | 30 | 8 (32%) M  17 (68%) F | 100% oral corticosteroids |
| Aigner 2002[26] | 45 | 69 | Level IV | 95% | Core Decompression | 41 | 33 (89%) M  4 (11%) F | 100% idiopathic |
| Arlet 1988[27] | 21 | 39 | Level IV | 40% | CD alone (simple drilling) | n/a (17 – 63 range) | 6 (35%) M  11 (65%) F | 82.3% Corticosteroids, 11.7% Alcohol, 5.8% idiopathic, |
| Beckmann 2013[28] | 12 | 13 | Level III | 90% | CD with 6 mm Trephine | 36 | 8 (66%) M  4 (34%) F | N/A |
| Beltran 1990[29] | 34 | 23 | Level IV | 80% | CD (Simple Drilling) | 39 | 15 (60%) M  10 (40%) F | 91.6% Corticosteroids, 8.4% idiopathic, |
| Bi 2019[30] | 36 | 26 | Level III | 70% | CD with trephine of 10mm | 35 | 15 (75%) M, 5 (25%) F | N/A |
| Bozic 1999[31] | 54 | 120 | Level IV | 100% | CD with Simple Drilling 8 mm Trephine | 38 | 21 (62%) M  13 (38%) F | 68.5% corticosteroid, 14.8% alcohol,12.9% Idiopathic |
| Chen 2016[32] | 42 | 6 | Level IV | 100% | CD with 3.5 mm solid core drill | 38 | 26 (62%) M  16 (38%) F | 50% corticosteroid, 31% alcohol, 19% idiopathic |
| Classen 2015[33] | 72 | 31 | Level IV | 95% | CD with cannulated 9 mm drill | 47 | 43 (72%) M  17 (28%) F | 12% corticosteroid, 10% alcohol, 12% nicotine, 45% idiopathic |
| Cruzpardos 2016[34] | 19 | 64 | Level III | 100% | Core Decompression | 37 | 14 (74%) M  5 (26%) F | 31.6% Corticosteroids, 26.3% Alcohol, 31.6% Idiopathic |
| Etemadifar 2014[35] | 22 | 12 | Level IV | 75% | multiple small­ diameter drilling | 45 | 9 (41%) M  13 (59%) F | 54.5% corticosteroids, 18% had history of drug use, 31.8% idiopathic |
| Fairbank 1995[36] | 128 | 132 | Level IV | 90% | Simple CD with the "Ficat coring device” | 40 | 45 (50%) M  45 (50%) F | 61% corticosteroid, 21% alcohol, 9% idiopathic |
| Gangji 2011[37] | 24 | 60 | Level II | 95% | Simple CD | 44 | 9 (47%) M  10 (53%) F | 84% corticosteroid, 8% alcohol, 8% idiopathic |
| Haberal 2019[38] | 30 | 34 | Level IV | 75% | CD multiple drilling, 2.5 mm Kirschner wire | 43 | 14 (64%) M, 8 (36%) F | 100% - solid organ transplantation |
| Hauzeur 2017[39] | 23 | 24 | Level I | 85% | Core Decompression | 50 | 13 (68%) M  6 (32%) F | 56% Corticosteroids, 30% Alcohol, 14% Idiopathic |
| Hernigou 2018[40] | 125 | 300 | Level III | 100% | a 3 mm diameter trephine (trocar of Mazabraud, Collin, France) | 36 | 78 (62%) M  47 (38%) F | 100% corticosteroids |
| Iorio 1998[41] | 33 | 63 | Level IV | 100% | CD with Simple Drilling 5, 8, 10 mm Trephine | 41 | 21 (91%) M  2 (9%) F | 58% Corticosteroids, 9% trauma, 3% alcohol, 24% idiopathic |
| Israelite 2005[42] | 316 | 68 | Level III | 90% | Core Decompression | N/A | N/A | N/A |
| Ito 2003[43] | 90 | 108 | Level II | 82% | Core Decompression | 33 | 18(24%) M  59 (76%) F | 75% Corticosteroids, 16.6% alcohol ,7.7% idiopathic |
| Kang 2011[44] | 60 | 63 | Level II | 82% | multiple small-diameter drilling | 46 | 35 (67%) M,  17 (32.7%) F | 59.8% Corticosteroids, 37.3% alcohol ,2.8% idiopathic |
| Kang 2018[45] | 53 | 48 | Level III | 90% | CD with Trephine over 2.0-mm K-wires | 47 | 38 (76%) M, 12 (24%) F | 24 (45%) idiopathic, 19 (36%) alcohol, 5 (9%) corticosteroids |
| Kristensen 1991[46] | 18 | 36 | Level IV | 75% | CD with Simple Drilling 4 mm Trephine | 53 | 7 (50%) M  7 (50%) F | 100% Idiopathic |
| Lakshminarayana 2019[47] | 36 | 54 | Level II | 55% | CD (8 mm) reamer of dynamic hip screw | 30 | N/A | N/A (no separate data for CD only) |
| Lausten 1990[48] | 30 | 18 | Level II | 90% | slightly conical 3-mm cannula | 40 | 25 (89%) M  3 (10.7%) F | 23.3% Corticosteroids, 36.6% alcohol ,20% idiopathic |
| Learmonth 1990[49] | 41 | 31 | Level IV | 90% | CD with 10 mm Trephine | 37 | 21 (65%) M  11 (35%) F | 22% corticosteroids, 50% alcohol |
| Li, Je 2017[50] | 55 | 54 | Level IV | 100% | multiple small-diameter using a 3‑mm Kirschner wire | 37 | 18 (53%) M  16 (47%) F | 33.6% Corticosteroids, 43.8% Alcohol, 22.4% Idiopathic |
| Maniwa 2000[51] | 26 | 94 | Level IV | 75% | 8-mm trephining device | 46 | 9 (47.3%) M  10 (52.7%) F | 46.1% Corticosteroids, 34.6% idiopathic, 3.8% Alcohol, 3.8% trauma |
| Markel 1996[12] | 54 | 27 | Level IV | 55% | 12-mm drill | 39 | 12 (26.6%) M,  33 (73.3%) F | 44.4% Corticosteroids, 31.1% SLE, 15.5% trauma/idiopathic, 11.1% Alcohol |
| Mazières 1997[52] | 20 | 24 | Level IV | 90% | Core Decompression | 43 | 16(88.8%) M  2 (11.1%) F | 38.8% Corticosteroids,  44.4% Alcohol,  16.6% Both alcohol +Corticosteroids |
| Miao 2015[53] | 34 | 26 | Level II | 81% | multiple 3.2-mm drill | 35 | 13 (43.3%) M  17 (56.7%) F | 60% Corticosteroids  27.5% idiopathic  12.5% Alcohol |
| Miyahara 2018[54] | 30 | 6 | Level IV | 65% | CD with cannulated 10-mm trephine | 44 | 11 (61%) M, 7 (39%) F | 9 (50%) corticosteroids, 4 (22%) idiopathic |
| Mohanty 2016[55] | 33 | 24 | Level III | 80% | multiple 4.5+3.5-mm drill | 37 | 17 (70.8%) M  7 (29.2%) F | 17.8 % Corticosteroids  13.3 % Alcohol  68.9% Idiopathic |
| Mont 1997[56] | 79 | 144 | Level IV | 85% | 8 to 10 mm Ficat biopsy cannula | 34 | 22 (44%) M  28 (56%) F | 100 % Corticosteroids  Out of which: 39.2% - SLE |
| Mont 1998[57] | 68 | 144 | Level IV | 95% | 8 to 10 mm Ficat biopsy cannula | 34 | 32 (61.5%) M  20 (38.5%) F | Corticosteroids – 59%  Alcohol – 20.5%,  Idiopathic – 20.5% |
| Mont 2004[58] | 45 | 24 | Level IV | 95% | multiple 3-mm Steinman pin | 42 | 13 (36.1%) M  23 (63.9%) F | Corticosteroids – 59.5%  Alcohol – 11.9%  Smoking- 26.1%  SLE-23.8% |
| Nori 2015[59] | 40 | 12 | Level IV | 45% | 8 mm Michele trephine | N/A | 16 (6.6%) M  8 (33.3%) F | 66.6% Corticosteroids  25% idiopathic  8.3% Alcohol |
| Pepke 2016[60] | 14 | 24 | Level I | 73% | 2.0 mm K-wires were drilled & then 5 mm trephine | 44 | 12 (85.7%) M  2 (14.3%) F | 16% corticosteroids  8.3% chemotherapy |
| Powell 1997[61] | 34 | 48 | Level IV | 70% | 7-mm trephining device | 35 | 10 (45.5%) M  12 (54.5%) F | 81.8% corticosteroids  9% Alcohol  4.5% Idiopathic |
| Sadile 2017[62] | 41 | 46 | Level II | 70% | 6 mm + 11 mm cannulated drill bits & 10mm trephine | 44 | 25 (71.4%) M  10 (28.5%) F | 21.2% corticosteroids,15.1% Alcohol, 39.3% Idiopathic |
| Sallam 2017[63] | 38 | 94 | Level III | 70% | Cannulated 8 mm drill | 33 | 32 (52.4%) M  29 (47.5%) F | 23.6% corticosteroids, Liver cirrhosis 18.4%, Hyperlipidemia 15.7%, 28.9% Idiopathic |
| Simank 1999[64] | 94 | 72 | Level IV | 70% | either a 6 mm drill or an 8 mm trephine | 40 | 57 (77%) M  17 (23%) F | Severe underlying disease was present in 67% (NOS) |
| Simank 2001[13] | 55 | 72 | Level III | 60% | 2 or 3 tracks using a 6-mm drill, or single track using an 8-mm trephine | 40 | 42 (76.5%) M  13 (23.5%) F | N/A |
| Song 2007[65] | 163 | 87 | Level IV | 100% | multiple drilling using 9/64-inch Steinmann pins | 36 | 120 (88.2%) M  16 (19.8%) F | 43.5% Corticosteroids, 36.8% idiopathic, 19.6% Alcohol, |
| Tabatabaee 2015[66] | 14 | 24 | Level I | 85% | Multiple 2.7 mm drilling | 29 | 10 (71%) M,  4 (29%) F | 67.5 % Corticosteroids, 32% idiopathic |
| Tooke 1988[67] | 45 | 36 | Level IV | 80% | Core Decompression | 40 | 14 (42.4%) M  19 (57.5%) F | 72.7% Corticosteroids 18.1% Alcohol, 9% idiopathic, |
| Yan 2015[68] | 42 | 26 | Level III | 75% | 4.5-mm diameter drill | 38 | 20 (49%) M  22 (51%) F | 66.5 % Corticosteroids, 33.5% idiopathic |
| Yin 2016[69] | 26 | 36 | Level III | 95% | multiple 3.0 mm Steinman pin drilling | 42 | 13 (81%) M  3 (19%) F | 10% Corticosteroids, 41% Alcohol, 48.2% idiopathic |
| Yoon 2000[70] | 39 | 61 | Level IV | 95% | 3–6 multiple cores using a 6.5-mm drill | 47 | 31 (100%) M  0 (0%) F | 25.6% Corticosteroids 51.2% Alcohol, 23% idiopathic, |
| *Totals and Averages* | **2540** | **75.1 (weighted mean)** | **-** | **82%** | **-** | **39** | **1122 M (61.5%)**  **702 F (38.5%)** | 53.5% Corticosteroids;  23.1% idiopathic and 22.5% alcohol abuse |
| Nr – number of hips; M – male; F – Female; Avg – average; N/A – not available, SLE – systemic lupus erythematosus, CD – core decompression. | | | | | | | | |
