# Supplementary material for: What are the outcomes of core decompression without augmentation in patients with nontraumatic osteonecrosis of the femoral head?
Source: Int Orthop. 2020 Sep 4;45(3):605–13. doi: 10.1007/s00264-020-04790-9 (PMC7892522; doi:10.1007/s00264-020-04790-9)
Supplement: Supplementary file 3 — (DOCX 31 kb) [file 264_2020_4790_MOESM3_ESM.docx]

**Supplementary Table 3.** Outcome of studies using the ARCO Classification

| **Study** | **Nr.** | **Avg. Follow-up** | **Preoperative Staging** | **Clinical assessment tool** | **Postoperative Clinical improvement** | **Time to clinical deterioration (Avg, months)** | **Radiographic success (no progression)** | **Time to THA, months (% of total hips)** |
| --- | --- | --- | --- | --- | --- | --- | --- | --- |
| Aigner 2002[26] | 45 | 69 | Stage I: 67% Stage II: 20%  Stage III: 13% | HHS | Stage I: YES Stage II: YES Stage III: NO | 16 | (% out of ARCO Pre-Op Sub-groups): Stage I: 97% Stage II: 45% Stage III: 0% | Stage II: 46.5 (44%)  Stage III: 16 (50%) |
| Beckmann 2013[28] | 12 | 13 | Stage II: 100% | HHS WOMAC VAS  SF36 – Physical  SF36- Mental | HHS -YES WOMAC-NO VAS-YES  SF36 – Physical - YES  SF36- Mental - YES | n/a | 83% | n/a |
| Bi 2019[30] | 36 | 26 | Stage II: 100% | HHS  VAS | HHS – Yes;  VAS - Yes | 26 | 72% (10/36 showed progression) | 26 (28%) |
| Gangji 2011[37] | 24 | 60 | Stage I:18% Stage II:82% | Lequesne Index VAS  WOMAC | Lequesne Index-YES VAS -YES  WOMAC-YES | no clinical deterioration | 27% | 26.5 (27%) |
| Haberal 2019[38] | 30 | 34 | Stage I: 13%  Stage 2A: 10%  Stage 2B: 7%  Stage 2C: 17%  Stage 3A: 10%  Stage 3B: 3%  Stage 3C: 33%  Stage 4: 7% | HHS  VAS | HHS – NO | n/a | n/a | n/a (9%) |
| Hauzeur 2017[39] | 23 | 24 | Stage I: 55.2%  Stage II: 44.8% | HHS WOMAC VAS | HHS- YES WOMAC-YES VAS-YES | no clinical deterioration | Evolution to ARCO stage IV was noted in 43% after 9.3 ± 2.2 months | 8.2 (65.5%) |
| Kang 2018[45] | 53 | 48 | Stage I: 2%  Stage II: 55%  Stage III: 36%  Stage IV: 7% | VAS | n/a | n/a | 59% (33/53 no progression) | 48 (49%) |
| Pepke 2016[60] | 14 | 24 | Stage II: 100% | HHS VAS | HHS- YES VAS-YES | n/a | (% out of ARCO Pre-Op Sub-groups): Group I – 43% Group II- 36% | n/a (37.5%) |
| Tabatabaee 2015[66] | 14 | 24 | Stage I: 14.2% Stage II: 50% Stage III: 35.7% | WOMAC VAS | WOMAC - YES VAS - YES | n/a | n/a | n/a (21.4%) |
| Yan 2015[68] | 42 | 26 | Stage I: 5.8% Stage II: 94.1% | HHS VAS | HHS-YES VAS-YES | 12 | N/a | 12 (9.5%) |
| Yin 2016[69] | 26 | 36 | Stage I: 9% Stage IIA:3% Stage II B: 32% Stage II C:50% Stage IIIA:6% | HHS VAS | HHS-YES VAS-YES | 22 | 42% -progressed | 14 (26.9%) |
| Nr – number of hips; Avg – average; THA – total hip replacement; n/a – not available; HHS – Harris Hip Score; VAS – visual analogue scale. | | | | | | | | |
