# Supplementary material for: What are the outcomes of core decompression without augmentation in patients with nontraumatic osteonecrosis of the femoral head?
Source: Int Orthop. 2020 Sep 4;45(3):605–13. doi: 10.1007/s00264-020-04790-9 (PMC7892522; doi:10.1007/s00264-020-04790-9)
Supplement: Supplementary file 4 — (DOCX 26 kb) [file 264_2020_4790_MOESM4_ESM.docx]

**Supplementary Table 4.** Outcome of studies using the Steinberg Classification

| **Study** | **Nr.** | **Avg. Follow-up** | **Preoperative Staging** | **Clinical assessment tool** | **Postoperative Clinical improvement** | **Time to clinical deterioration (Avg, months)** | **Radiographic success (no progression)** | **Time to THA, months (% of total hips)** |
| --- | --- | --- | --- | --- | --- | --- | --- | --- |
| Classen 2015[33] | 72 | 31 | Stage I+II+III:100% | HHS VAS | HHS -YES VAS-YES | n/a | (% out of Steinberg Pre-Op Sub-groups): Stage 1: 89% Stage 2: 67% Stage 3: 53% | 10.5 (33%) |
| Etemadifar 2014[35] | 22 | 12 | Stage I: 36.4% Stage IIA: 63.6% | VAS  ROM | VAS-YES (100%)  ROM – YES (100%) | no clinical deterioration | (% out of Steinberg Pre-Op Sub-groups):  Stage I: 87.5% Stage IIA: 78.5  Stage IIB:13.6% % | n/a |
| Hernigou 2018[40] | 125 | 300 | Stage I: 55.2%  Stage II: 44.8% | HHS WOMAC VAS | HHS -YES WOMAC-YES VAS-YES | 60 months | 28% | n/a (76%) |
| Israelite 2005[42] | 316 | 68 | Stage I: 22.5% Stage II: 39% Stage III: 4.5% Stage IV: 33% Stage V: 1% | HHS | HHS -YES | 29 | n/a | 29 (38%) |
| Ito 2003[43] | 90 | 108 | Stage IA: 10% Stage IB: 14.4% Stage IC:18.8% Stage IIA: 6.6% Stage IIB: 13.3% Stage IIC: 24.4% Stage IIIA: 0% Stage IIIB: 3.3% Stage IIIC: 8.8% | HHS | HHS -YES | 108 months (62.2%) | 33.3% | 108 (38.8%) |
| Markel 1996[12] | 54 | 27 | Stage 0: 7.4%  Stage I: 12.9%  Stage IIA: 33.3%  Stage IIB: 22.2%  Stage IIC: 3.7%  Stage IIIA: 3.7%  Stage IVA: 9.2%  Stage IVB: 3.7%  Stage IVC: 1.8%  Stage VA: 1.8% | HHS | NO | 11 months | (% out of Steinberg Pre-Op Sub-groups)  Stage 0: 50%  Stage I: 42.8%  Stage IIA: 27.7%  Stage IIB: 50%  Stage IIC: 50%  Stage III: 50%  Stage IV: 12.5%  Stage V: 0 % | 11 (48.1%) |
| Miao 2015[53] | 34 | 26 | Stage I – 41.1% Stage II – 58.9% | HHS | YES | n/a | 76.4%- no progression | n/a (23.5%) |
| Simank 1999[64] | 94 | 72 | Steinberg 0/ I/ II: 50% Steinberg III/ IV/ V:50% | Merle d'Aubigné-Postel | n/a | n/a | (% out of Steinberg Pre-Op Sub-groups): Steinberg 0/ I/ II: 57% Steinberg III/ IV/ V: 39% Overall 52%- deterioration | n/a (31.9%) |
| Simank 2001[13] | 55 | 72 | Steinberg stages 0/I/II:58.2%  Steinberg stages III/IV/ V: 41.8% | n/a | n/a | n/a | Overall survival rate – 56% | n/a (44%) |
| Nr – number of hips; Avg – average; THA – total hip replacement; n/a – not available; HHS – Harris Hip Score; VAS – visual analogue scale. | | | | | | | | |
