# Supplementary material for: What are the outcomes of core decompression without augmentation in patients with nontraumatic osteonecrosis of the femoral head?
Source: Int Orthop. 2020 Sep 4;45(3):605–13. doi: 10.1007/s00264-020-04790-9 (PMC7892522; doi:10.1007/s00264-020-04790-9)
Supplement: Supplementary file 5 — (DOCX 39 kb) [file 264_2020_4790_MOESM5_ESM.docx]

**Supplementary Table 5.** Outcome of studies using the Ficat Classification

| **Study** | **Nr.** | **Avg. Follow-up** | **Preoperative Staging** | **Clinical assessment tool** | **Postoperative Clinical improvement (Percentage)** | **Time to clinical deterioration (Avg, months)** | **Radiographic success (no progression)** | **Time to THR, months (%)** |
| --- | --- | --- | --- | --- | --- | --- | --- | --- |
| Aaron 1989[24] | 50 | 38 | Stage II:52% Stage III:48% | Merle d'Aubigné-Postel | Stage II: YES (62%) Stage III: YES (25%) | n/a | (% out of FICAT Pre-Op Subgroups): Stage II: 62% improved; 38% didn't deteriorate Stage III: 25% improved; 33% didn't deteriorate | 26 (42%) |
| Arlet 1988[27] | 21 | 39 | Stage I & II | n/a | n/a | n/a | 40% | 9.2 (40%) |
| Beltran 1990[29] | 34 | 23 | Stage I: 64.7% Stage II: 35.3% | n/a | n/a | n/a | 43% collapse with 25-50% preop infarction and 87% collapse with >50% preop infarction. | 6.7 (72.7%) |
| Chen 2016[32] | 42 | 6 | Stage I + II: 79% Stage III: 21% | HHS Pain assessment index (PRI) | (HHS): Stage I+II: YES Stage III: YES (PRI): Stage I+II: YES Stage III: NO | no clinical deterioration | 11.9% | n/a |
| Cruzpardos 2016[34] | 19 | 64 | Stage I: 31.2% Stage II: 68.8% | Merle d'Aubigné-Postel VAS, ROM | Yes | no clinical deterioration | 47.3% | 35 (42.1%) |
| Fairbank 1995[36] | 128 | 132 | Stage I – 19.5% Stage II – 39.8% Stage III –40.6% | Merle d'Aubigné-Postel VAS | n/a | n/a | Overall 43%  (% out of FICAT Pre-Op Sub-groups): Stage I: 78%  Stage II: 38%  Stage III:7% | 55 (38%) |
| Iorio 1998[41] | 33 | 63 | Stage I: 21.2% Stage IIA: 60.6% Stage IIB: 18.1% | VAS | Yes | 19.2 M (52%) | 45%(21.6 M) | 63 (33%) |
| Kristensen 1991[46] | 18 | 12-60 | Stage I: 100% | VAS | Yes (100%) | 12 months (66.7%) | 77.7% | n/a |
| Lausten 1990[48] | 30 | 18 | Stage I: 38% Stage II: 38% Stage III: 24% | HHS  intraosseous baseline pressure | Stage I: YES Stage II: No Stage III: No | Stage I: 27.2% (5 M) Stage II: 54% (9 M) Stage III: 57% (10 M) | Stage I: 73% (5 M) Stage II: 63.6% (9 M) Stage III: 57% (10 M) | 12-24 (51.7%) |
| Learmonth 1990[49] | 41 | 31 | Stage I: 29.2%  Stage II: 70.8% | HHS  intraosseous baseline pressure | Stage I: YES (42%)  Stage II: YES (17%) | 18 | (% out of FICAT Pre-Op Sub-groups): Stage I: 25% Stage II:14% | 18 (44%) |
| Li, Je 2017[50] | 55 | 54 | Stage I= 31%  Stage II= 69% | HHS | Stage I: YES   Stage II: YES | n/a | Stage I: 88% (12 M)  Stage II; 71% (12 M) | n/a |
| Maniwa 2000[51] | 26 | 94 | Stage I – 38.4% Stage II – 61.6% | Merle d'Aubigné-Postel | YES | n/a | 23%-THA  30.7%- further intervention | n/a (23%) |
| Mazières 1997[52] | 20 | 24 | Stage II: 100% | n/a | n/a | n/a | 15% - improvement 35% - no progression | n/a (45%) |
| Mont 1997[56] | 79 | 144 | Stage I - 16% Stage II – 44% Stage III – 40% | n/a | n/a | n/a | FICAT- % of success: Stage I - 87.5% Stage II – 36.3% Stage III – 25% | n/a (46.8%) |
| Mont 1998[57] | 68 | 144 | Stage III - 100% | HHS | YES | n/a | (% out of FICAT Pre-Op Sub-groups) Stage III :59% Stage IV: 8% | n/a (58.8%) |
| Mont 2004[58] | 45 | 24 | Stage I: 66.6% Stage II: 33.3% | Modified HHS | 24 (80%) Stage I hips and 8 Stage II hips (57%) improved | n/a | (% out of FICAT Pre-Op Sub-groups) Stage I: 80% Stage II: 53.3% | n/a (28.8%) |
| Nori 2015[59] | 40 | 12 | Stage I & II | HHS | I-YES II–YES | 12 | n/a | 12 (15%) |
| Powell 1997[61] | 34 | 48 | Stage o: 8.8% Stage I: 58.8% Stage II:32.3% | HHS | YES | n/a | (% out of FICAT Pre-Op Sub-groups) Stage 0: 100% Stage I: 65% Stage II:9.1% | n/a (31%) |
| Sadile 2017[62] | 41 | 46 | Stage I: 19.5% Stage II: 80.5% | n/a | n/a | 13.4 | n/a | 13.4 (63.4%) |
| Tooke 1988[67] | 45 | 36 | Stage I: 22.2% Stage II:57.7% Stage III:20% StageIV:0% | UCLA scoring | No | 14 | (% out of FICAT Pre-Op Sub-groups): Stage I: 100% Stage II:57.7% Stage III:44.5% | 14 (42.3%) |
| Nr – number of hips; Avg – average; THR – total hip replacement; n/a – not available; HHS – Harris Hip Score; VAS – visual analogue scale. | | | | | | | | |
