# Supplementary material for: What are the outcomes of core decompression without augmentation in patients with nontraumatic osteonecrosis of the femoral head?
Source: Int Orthop. 2020 Sep 4;45(3):605–13. doi: 10.1007/s00264-020-04790-9 (PMC7892522; doi:10.1007/s00264-020-04790-9)
Supplement: Supplementary file 6 — (DOCX 29 kb) [file 264_2020_4790_MOESM6_ESM.docx]

**Supplementary Table 6.** Outcome of studies using the Modified Ficat Classification

| **Study** | **Nr.** | **Avg. Follow-up** | **Preoperative Staging** | **Clinical assessment tool** | **Postoperative Clinical improvement** | **Time to clinical deterioration (Avg, months)** | **Radiographic success (no progression)** | **Time to THR, months (% of total hips)** |
| --- | --- | --- | --- | --- | --- | --- | --- | --- |
| Abrisham 2013[25] | 37 | 24 | Stage I – 27% Stage IIA – 73% | n/a | n/a | n/a | 25% - no progression Stage I – 0%  Stage IIA – 48.1% | n/a |
| Bozic 1999[31] | 54 | 120 | Stage I: 24% Stage IIA: 42.5% Stage IIB: 18.5% Stage III: 14.8% | n/a | Overall 48%  Stage I: 92% Stage IIA: 52% Stage IIB: 20% Stage III: 0% | 97 M | (% out of FICAT Pre-Op Sub-groups): Stage I: 69.2% Stage IIA: 43.4% Stage IIB: 10% Stage III: 0% | 22 M (63%) |
| Kang 2011[44] | 60 | 63 | Stage IIA: 28.9% Stage IIB: 46.1% Stage III: 25% | HHS | 71.2% | 81.3 M | 67.4% | 62.5  (overall - 28.8%;  Stage II - 21%;  Stage III - 54%) |
| Lakshminarayana 2019[47] | 36 | 54 | Stage I : 100% | HHS  VAS | HHS – YES;  VAS - YES | n/a | 75% (9/36 showed radiographic progression) | n/a |
| Markel 1996[12] | 54 | 27 | Stage I -20.3%  Stage II A– 59.2%  Stage IIB– 12.9%  Stage III -7.4% | HHS | NO | 11.1 | (% out of FICAT Pre-Op Sub-groups)  Stage I -45.4%  Stage II A–37.5 %  Stage IIB–14.3 %  Stage III -25%% | 11.1 (48.1%) |
| Miyahara 2018[54] | 30 | 6 | Stage I 37%  Stage 2A 63% | Merle d’Aubigne and Postel | Only partial improvement | n/a | 27% (22/30 have collapsed) | 6 (50%) |
| Mohanty 2016[55] | 33 | 24 | Stage I - 12.2% Stage IIA – 54.6% Stage IIB – 9% Stage III – 24.2% | HHS | Stage I- YES Stage IIA – YES Stage IIB – YES Stage III – NO | n/a | 54.5 %- no progression | n/a (27%) |
| Sallam 2017[63] | 38 | 94 | Stage I: 10.5% Stage IIA: 44.7% Stage IIB: 18.4% Stage III: 26.3% | HHS | Stage I: 75% Stage IIA : 65% Stage IIB : 29% Stage III :5 0% | 51.8 | (% out of FICAT Pre-Op Sub-groups) Stage I: 50% Stage IIA:42.2% Stage II B: 28.6% Stage III:30% | 51.8 (38.2%) |
| Song 2007[65] | 163 | 87 | Stage I: 23.9% Stage IIA: 39.3% Stage IIB: 10.4% Stage III: 26.4% | HHS | N/a | 25.2 | (% out of FICAT Pre-Op Sub-groups): Stage I: 79.5% Stage IIA: 76.6% Stage IIB: 76.5% Stage III: 34.9% | 25.2 (30.6%) |
| Yoon 2000[70] | 39 | 61 | Stage I :43.5% Stage II:35.8% Stage III:20.5% | N/a | N/a | 48.7 | (% out of FICAT Pre-Op Sub-groups): Stage I :70.6% Stage II:35.8% Stage III: 0% | 18 (48.7%) |
| Nr – number of hips; Avg – average; THR – total hip replacement; n/a – not available; HHS – Harris Hip Score; VAS – visual analogue scale. | | | | | | | | |
