# Supplementary material for: What are the outcomes of core decompression without augmentation in patients with nontraumatic osteonecrosis of the femoral head?
Source: Int Orthop. 2020 Sep 4;45(3):605–13. doi: 10.1007/s00264-020-04790-9 (PMC7892522; doi:10.1007/s00264-020-04790-9)
Supplement: Supplementary file 7 — (DOCX 41 kb) [file 264_2020_4790_MOESM7_ESM.docx]

**Supplementary Table 7.** Average percentage and time to total hip replacement (THR)

| Study | Preoperative Staging | No. of hips | Average Follow-up (months) | Average Time to THR (months) | Percentage of hips undergoing THR (%) |
| --- | --- | --- | --- | --- | --- |
| Aigner 2002[26] | **ARCO**  Stage I: 67% Stage II: 20%  Stage III: 13% | 45 | 69 | 46.5 | 7/45(15.5%) |
| Arlet 1988[27] | **Ficat**  Stage I & II: 100% | 21 | 39 | 9.2 | 8/21(38%) |
| Bi 2019[30] | **ARCO**  Stage II: 100% | 36 | 26 | 26 | 10/36(28%) |
| Classen 2015[33] | **Steinberg**  Stage I+II+III:100% | 72 | 31 | 10.5 | 24/72 (33%) |
| Cruzpardos 2016[34] | **Ficat**  Stage I: 31.2% Stage II: 68.8% | 19 | 64 | 35 | 8/19 (42%) |
| Gangji 2011[37] | **ARCO**  Stage I:18%  Stage II:82% | 11 | 60 | 26.5 | 3/11(27.2%) |
| Hauzeur 2017[39] | **ARCO**  Stage I: 55.2%  Stage II: 44.8% | 23 | 24 | 8.2 | 15/23 (65.2%) |
| Israelite 2005[42] | **Steinberg**  Stage I: 22.5%  Stage II: 39%  Stage III: 4.5%  Stage IV: 33%  Stage V: 1% | 276 | 68 | 29 | 104/276 (38%) |
| Kang 2011[44] | **Modified Ficat**  Stage IIA: 28.9%  Stage IIB: 46.1%  Stage III: 25% | 60 | 63 | 62.5 | 15/52 (29%) |
| Kang 2018[45] | **ARCO**  Stage I: 2%  Stage II: 55%  Stage III: 36%  Stage IV: 7% | 53 | 48 | 48 | 26/53 (49%) |
| Lausten 1990[48] | **Ficat**  Stage I: 38%  Stage II: 38%  Stage III: 24% | 29 | 18 | 7.8 | 15/29 (52%) |
| Learmonth 1990[49] | **Ficat**  Stage I: 29.2%  Stage II: 70.8% | 41 | 31 | 18 | 18/41 (44%) |
| Markel 1996[12] | **Steinberg**  Stage 0: 7.4%  Stage I: 12.9%  Stage IIA: 33.3%  Stage IIB: 22.2%  Stage IIC: 3.7%  Stage IIIA: 3.7%  Stage IVA: 9.2%  Stage IVB: 3.7%  Stage IVC: 1.8%  Stage VA: 1.8% | 54 | 27 | 11.1 | 26/54 (48%) |
| Sadile 2017[62] | **Ficat**  Stage I: 19.5%  Stage II: 80.5% | 41 | 46 | 13.4 | 26/41 (63%) |
| Sallam 2017[63] | **Modified Ficat**  Stage I: 10.5%  Stage IIA: 44.7%  Stage IIB: 18.4%  Stage III: 26.3% | 38 | 94 | 51.8 | 13/34 (38.2%) |
| Song 2007[65] | **Modified Ficat**  Stage I: 23.9%  Stage IIA: 39.3%  Stage IIB: 10.4%  Stage III: 26.4% | 163 | 87 | 25.2 | 50/163 (30.6%) |
| Tooke 1988[67] | **Ficat**  Stage I: 22.2%  Stage II:57.7%  Stage III:20% | 45 | 36 | 14 | 20/45 (42.3%) |
| Yan 2015[68] | **ARCO**  Stage I: 5.8%  Stage II: 94.1% | 42 | 26 | 12 | 4/42 (9.5%) |
| Yin 2016[69] | **ARCO**  Stage I: 9%  Stage IIA:3%  Stage II B: 32%  Stage II C:50%  Stage IIIA:6% | 26 | 36 | 14 | 7/26 (26.9%) |
| Yoon 2000[70] | **Modified Ficat**  Stage I :43.5%  Stage II:35.8%  Stage III:20.5% | 39 | 61 | 18 | 19/39 (48.7%) |
| TOTAL AND AVERAGES | **890/1134 (78.5%) of hips with “early stage avascular necrosis”:**  *Ficat (6 studies):*  Stage I and II – 180/196  Stage III and IV – 16/196  *Modified Ficat (4 studies)*  Stage I and II – 217/300  Stage III and IV – 83/300  *Steinberg (3 studies):*  Stage I + II +III: 299/402  Stage IV+V+VI: 103/402  *ARCO (7 studies):*  Stage I and II – 194/236  Stage III and IV – 42/236 | 1134 | 56 (weighted mean) | 26.3 (weighted mean) | 431/1134 (38%) |
| No. – number; THR – total hip replacement; ARCO – „Association Research Circulation Osseous” | | | | | |
