# Supplementary figures and images for: What are the outcomes of core decompression without augmentation in patients with nontraumatic osteonecrosis of the femoral head?
Source: Int Orthop. 2020 Sep 4;45(3):605–13. doi: 10.1007/s00264-020-04790-9 (PMC7892522; doi:10.1007/s00264-020-04790-9)

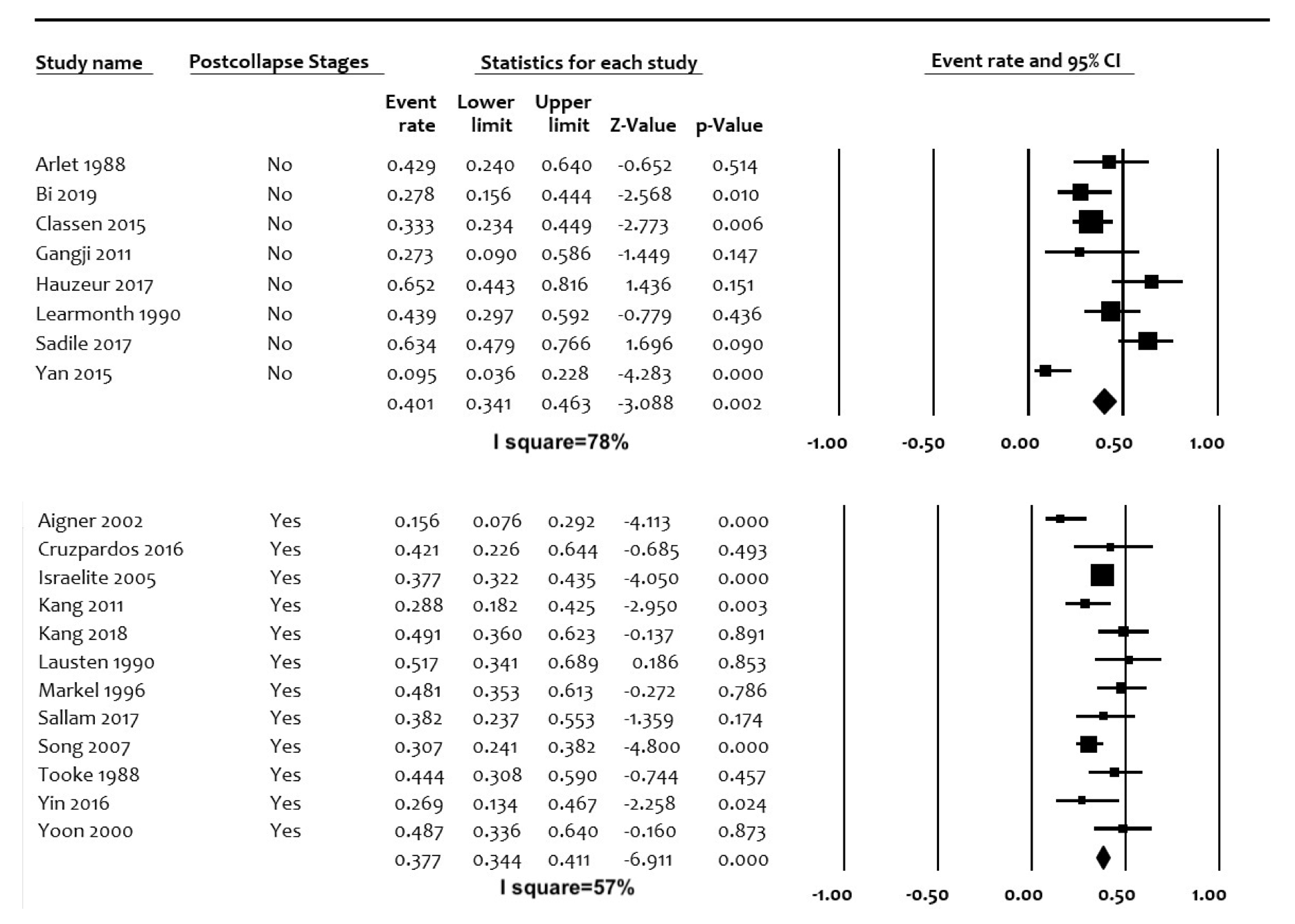

Supplement: Supplementary file 8 — Forest plot differentiating studies that included patients with preoperative post-collapse stages of osteonecrosis from those that did not. Event – conversion to THA (total hip arthroplasty). The size of the square represents the weight that the corresponding study exerts. I2 – value of calculated heterogeneity (JPG 727 kb) [file 264_2020_4790_MOESM8_ESM.jpg]

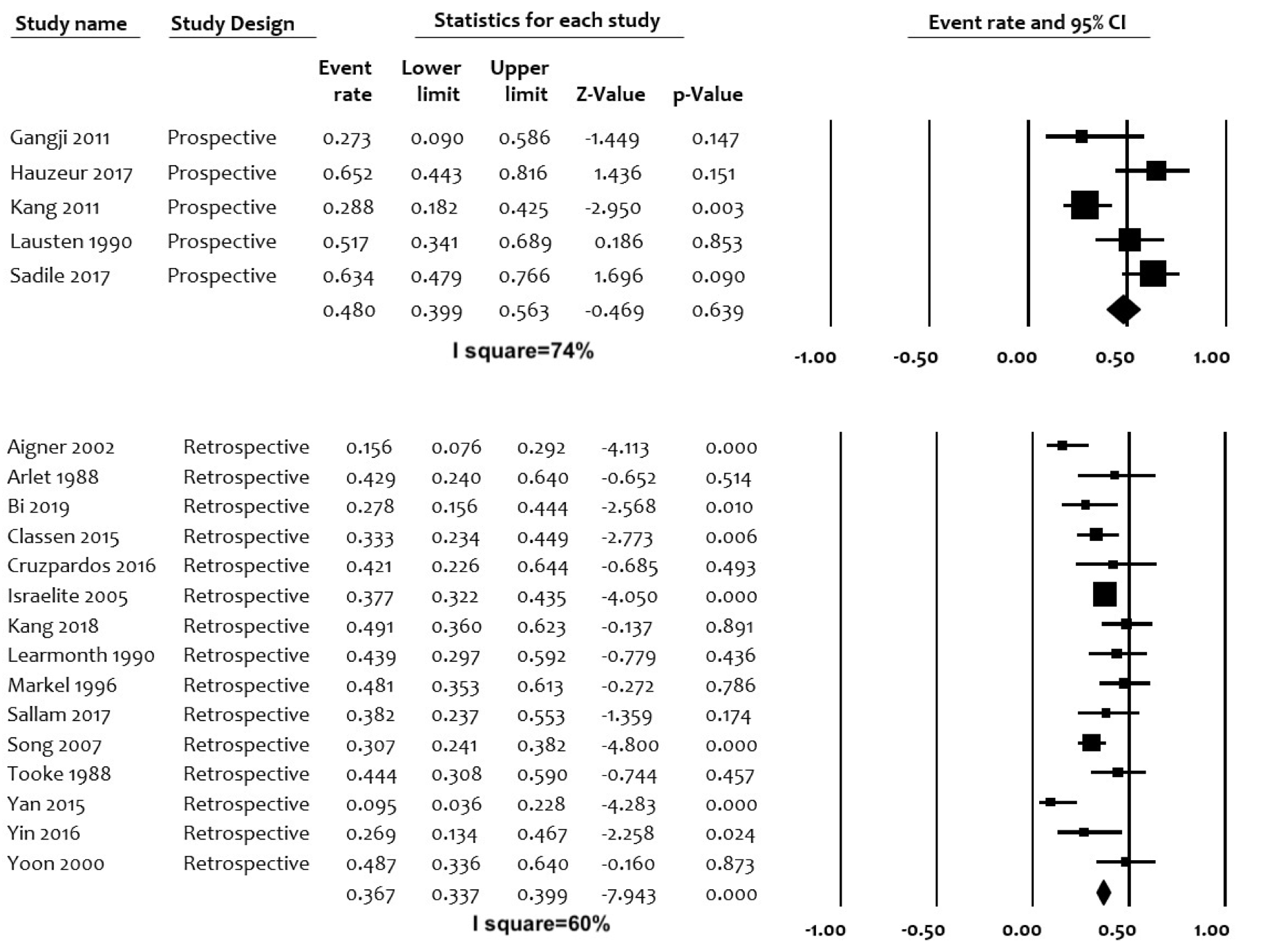

Supplement: Supplementary file 9 — Forest plot differentiating results from retrospective versus prospective studies. Event – conversion to THA (total hip arthroplasty). The size of the square represents the weight that the corresponding study exerts. I2 – value of calculated heterogeneity (JPG 841 kb) [file 264_2020_4790_MOESM9_ESM.jpg]
